# Supplementary figures and images for: Morphofunctional analysis of antigen uptake mechanisms following sublingual immunotherapy with beads in mice
Source: PLoS One. 2018 Dec 20;13(12):e0201330. doi: 10.1371/journal.pone.0201330 (PMC6301667; doi:10.1371/journal.pone.0201330)

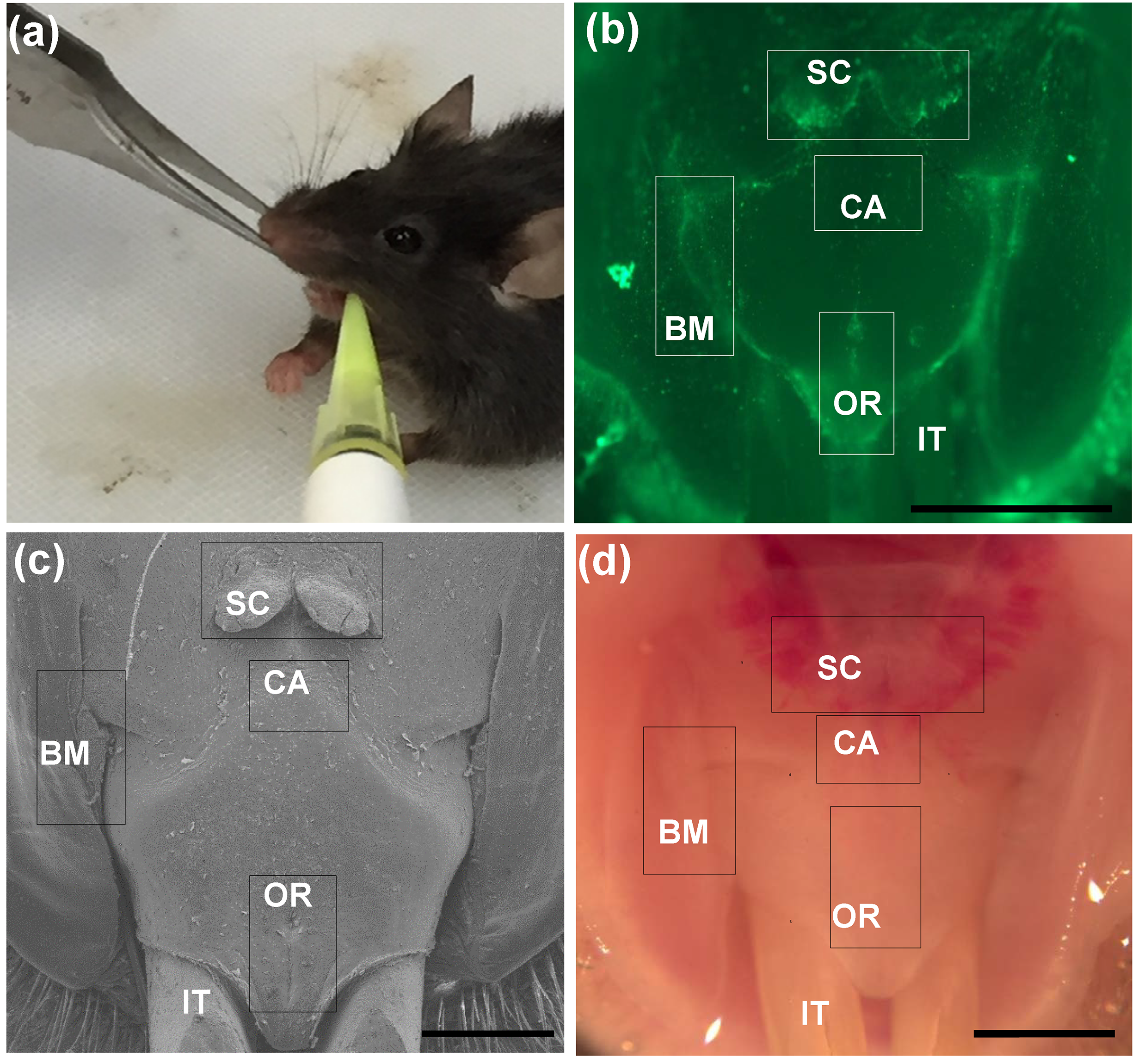

Supplement: S1 Fig — (a) Method of sublingual administration with a 20 μL micropipette of either PBS or beads to the lower part of the mouse’s tongue after raising the tongue. (b) Fluorescent stereomicroscopic image of the sublingual mucosa after cutting the anterior end of the tongue in the experimental group (latex beads, 3-month-old males). Notice fluorescently labelled bead accumulations in the sublingual caruncle (SC), buccal mucosa (BM), and oral rostral (OR) in front of the incisor teeth (IT) with no fluorescence detected in the negative control area (CA). (c) SEM image of the previous areas. (d) Fluorescent stereomicroscopic image of the sublingual mucosa in the control group. Notice the absence of fluorescent staining. Scale bar = 1 mm. (TIF) [file pone.0201330.s001.tif]

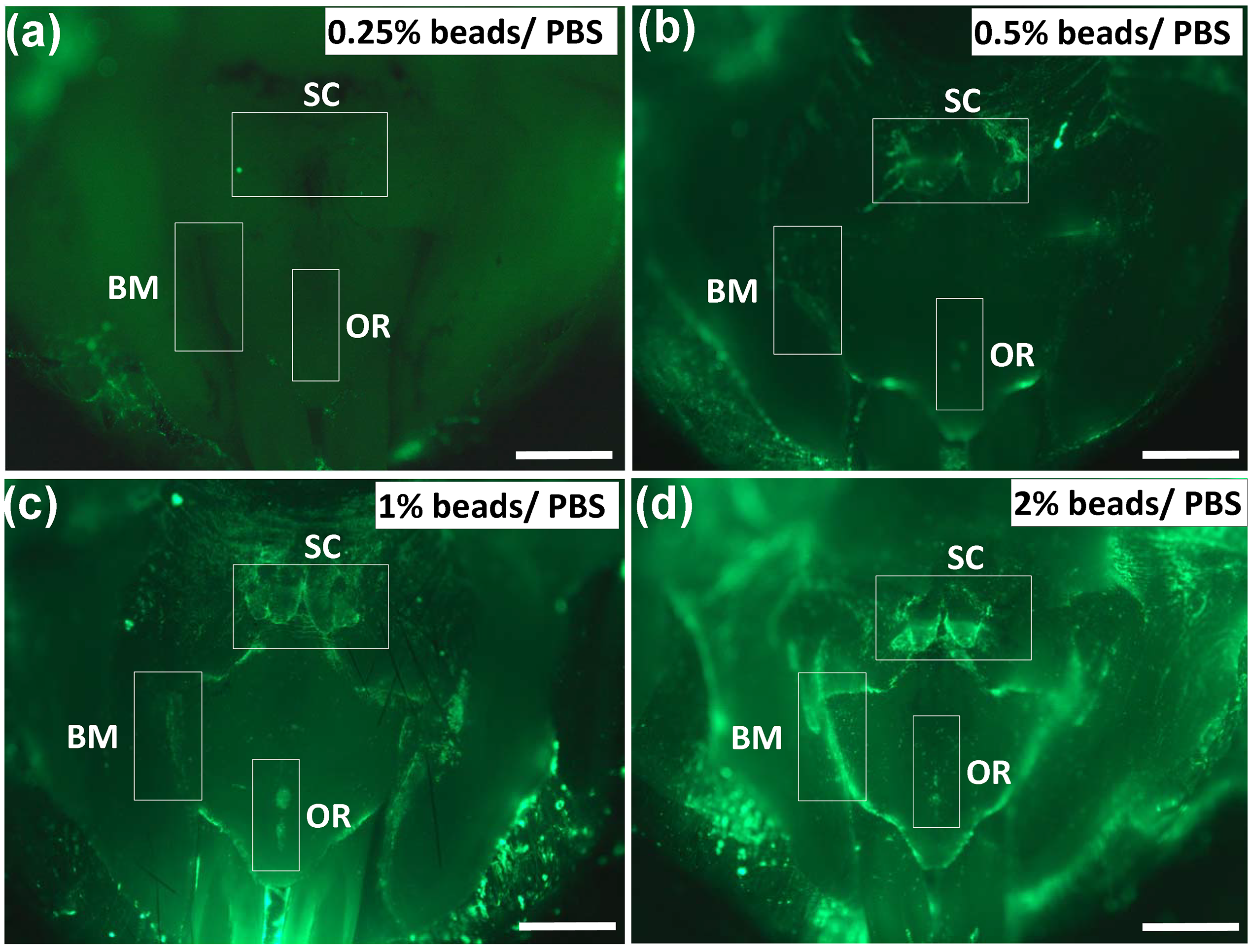

Supplement: S2 Fig — Notice fluorescently labelled bead accumulations in the sublingual caruncle (SC), buccal mucosa (BM), and oral rostral (OR) were rarely observed at 0.25% concentration (a); faintly observed at 0.5% concentration (b); clearly observed at 1% concentration (c); and over fluorescence illumination at 1% concentration (d). Scale bars = 30 μm. (TIF) [file pone.0201330.s002.tif]

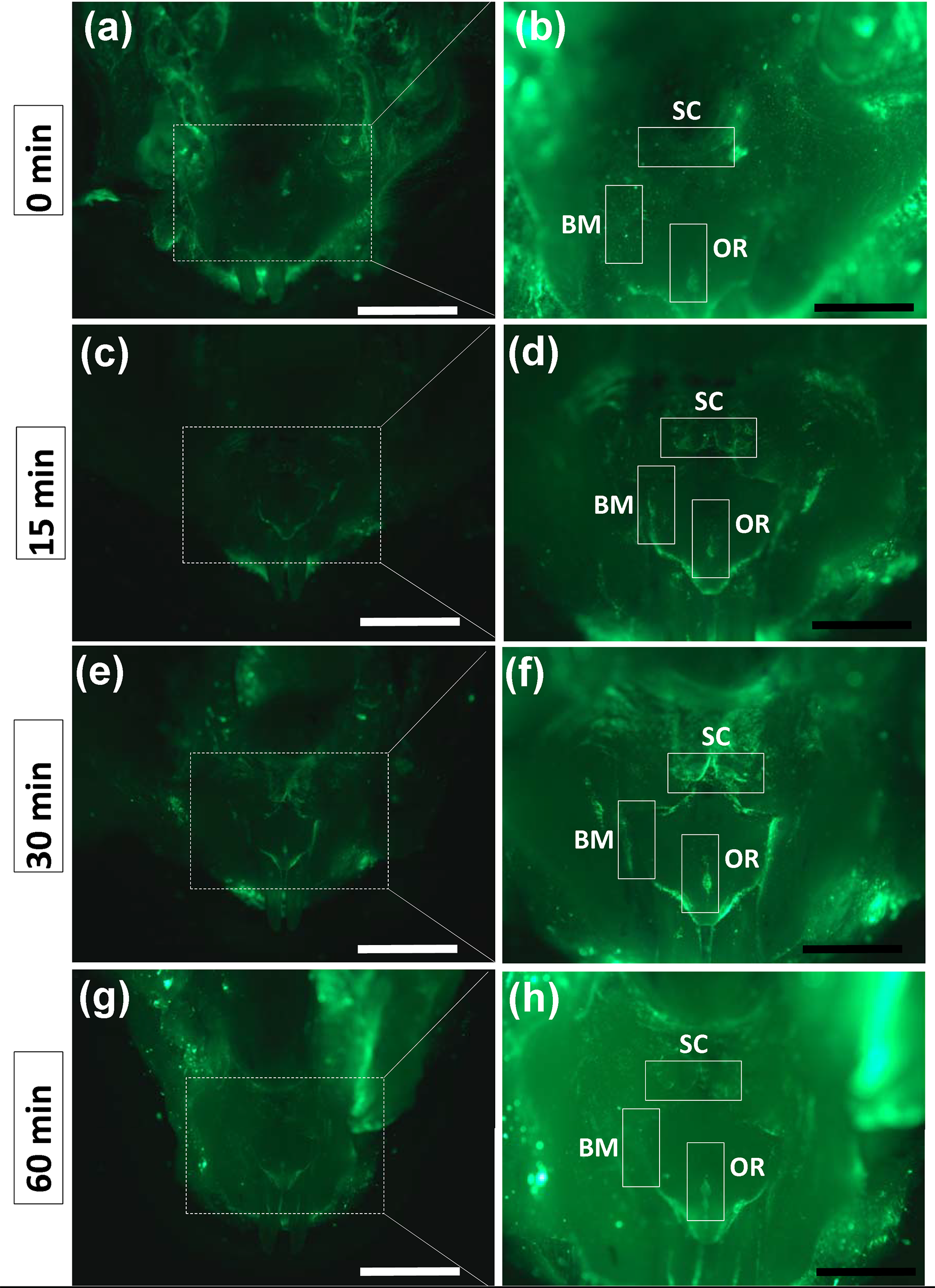

Supplement: S3 Fig — Notice fluorescently labelled beads were rarely observed at zero min (a, b); and (g, h); faintly observed at 15 min (c, d) and 60 min (b); clearly observed at 30 (e, f) min 1% concentration; and over fluorescence illumination at 1% concentration (d). Scale bars = 100 μm (a, c, e, g) and 40 μm (b, d, f, h). (TIF) [file pone.0201330.s003.tif]
